# Phase I clinical trial with IL-2-transfected xenogeneic cells administered in subcutaneous metastatic tumours: clinical and immunological findings

**DOI:** 10.1054/bjoc.2000.1492

**Published:** 2000-11-22

**Authors:** E Tartour, M Mehtali, X Sastre-Garau, I Joyeux, C Mathiot, J M Pleau, P Squiban, C Rochlitz, M Courtney, P Jantscheff, R Herrmann, P Pouillart, W H Fridman, T Dorval

**Affiliations:** Department of Tumor Biology, Institut Curie, Université Pierre et Marie Curie, Paris, France; Inserm U 255, Paris, France; Transgene, 11 Rue de Molsheim 67082, Strasbourg Cedex, France; Division of Medical Oncology, Kantonsspital, Basel, Switzerland; Department of Medical Oncology, Institut Curie, Paris, France

**Keywords:** xenogeneic cells, interleukin-2, T cell activation, cytokine subcutaneous metastasis

## Abstract

Various studies have emphasized an immunodepression state observed at the tumour site. To reverse this defect and based upon animal studies, we initiated a phase I clinical trial of gene therapy in which various doses of xenogeneic monkey fibroblasts (Vero cells) genetically engineered to produce human IL-2 were administered intratumorally in 8 patients with metastatic solid tumours. No severe adverse effect was observed in the 8 patients analysed during this clinical trial even in the highest dose (5 ¥ 107 cells) group. This absence of toxicity seems to be associated with rapid elimination of Vero-IL-2 cells from the organism. Indeed, exogenous IL-2 mRNA could no longer be detected in the peripheral whole blood 48 hours after Vero-IL-2 cell administration. In addition, we did not find any expression of exogenous IL-2 mRNA in post-therapeutic lesions removed 29 days after the start of therapy. A major finding of this trial concerns the two histological responses of two treated subcutaneous nodules not associated with an apparent clinical response. The relationship between local treatment and tumour regression was supported by replacement of tumour cells by inflammatory cells in regressing lesions and marked induction of T and natural killer cell derived cytokines (IL-2, IL-4, IFNg …) in post-therapeutic lesions analysed 28 days after the start of Vero-IL-2 administration. Gene therapy using xenogeneic cells as vehicle may therefore present certain advantages over other vectors, such as its complete absence of toxicity. Furthermore, the in vivo biological effect of immunostimulatory genes, i.e IL-2-, may be potentiated by the xenogeneic rejection reaction. © 2000 Cancer Research Campaign http://www.bjcancer.com


British Journal of Cancer (2000) 83(11), 1454–1461
© 2000 Cancer Research Campaign
doi: 10.1054/ bjoc.2000.1492, available online at http://www.idealibrary.com on IDEAL®

### Phase I clinical trial with IL-2-transfected xenogeneic cells administered in subcutaneous metastatic tumours: clinical and immunological findings

**E Tartour,[1,2] M Mehtali,[3] X Sastre-Garau,[1] I Joyeux,[1] C Mathiot,[1] JM Pleau,[1] P Squiban,[3] C Rochlitz,[4] M Courtney,[3] P Jantscheff,[4] R Herrmann,[4] P Pouillart,[5] WH Fridman[1,2] and T Dorval[5]**

[1]Department of Tumor Biology, Institut Curie, Université Pierre et Marie Curie, Paris, France; [2]Inserm U 255, Paris, France; [3]Transgene, 11 Rue de Molsheim 67082, Strasbourg Cedex, France; [4]Division of Medical Oncology, Kantonsspital, Basel, Switzerland; [5]Department of Medical Oncology, Institut Curie, Paris, France

Summary Various studies have emphasized an immunodepression state observed at the tumour site. To reverse this defect and based upon animal studies, we initiated a phase I clinical trial of gene therapy in which various doses of xenogeneic monkey fibroblasts (Vero cells) genetically engineered to produce human IL-2 were administered intratumorally in 8 patients with metastatic solid tumours. No severe adverse effect was observed in the 8 patients analysed during this clinical trial even in the highest dose (5 ¥ 107 cells) group. This absence of toxicity seems to be associated with rapid elimination of Vero-IL-2 cells from the organism. Indeed, exogenous IL-2 mRNA could no longer be detected in the peripheral whole blood 48 hours after Vero-IL-2 cell administration. In addition, we did not find any expression of exogenous IL-2 mRNA in post-therapeutic lesions removed 29 days after the start of therapy. A major finding of this trial concerns the two histological responses of two treated subcutaneous nodules not associated with an apparent clinical response. The relationship between local treatment and tumour regression was supported by replacement of tumour cells by inflammatory cells in regressing lesions and marked induction of T and natural killer cell derived cytokines (IL-2, IL-4, IFNg …) in post-therapeutic lesions analysed 28 days after the start of Vero-IL-2 administration. Gene therapy using xenogeneic cells as vehicle may therefore present certain advantages over other vectors, such as its complete absence of toxicity. Furthermore, the in vivo biological effect of immunostimulatory genes, i.e IL-2-, may be potentiated by the xenogeneic rejection reaction. © 2000 Cancer Research Campaign http://www.bjcancer.com

**Keywords:** xenogeneic cells; interleukin-2; T cell activation; cytokine subcutaneous metastasis

Although tumours express antigens recognized by cytotoxic T lymphocytes (CTL), they are mostly non-immunogenic, as they cannot activate T cells or maintain an activated T cell response (Speiser et al, 1997). To increase their immunogenicity, superantigens, haptens, foreign antigens derived from viruses, or allogeneic MHC genes have been introduced into tumours (Lindenmann and Klein, 1967; Fearon et al, 1988; Plautz et al, 1993; Stopeck et al, 1997; Berd et al, 1998; Dow et al, 1998; Heo et al, 1998). These modified tumours enhance immune response against wild-type tumour cells and clinical responses have been recorded. The identification of specific tumour antigens has led to the hypothesis that failure of the immune system to eliminate tumours resulted from an absence of a costimulatory signal provided by tumour cells which is thought to be required for full activation of T cells (Janeway and Bottomly, 1994; Chen, 1998). Various groups have shown that tumour cells transfected to express immunostimulatory cytokines are able to induce immune responses leading to rejection in experimental animal models (Fearon et al, 1990; Schmidt-Wolf and Schmidt Wolf, 1995; Tartour and Fridman, 1998). In some cases, vaccination with cytokine-producing tumour cells protected against subsequent challenge with an otherwise lethal dose of wild-type tumour and eliminated pre-existing cancer deposits (Gansbacher et al, 1990; Dranoff et al, 1993; Maass et al, 1995; Zatloukal et al, 1995; Clary et al, 1997). Unfortunately, in man, the development of autologous tumour vaccines has proven to be more complex due to the requirement for establishing tumour cell lines for each patient and the need for efficient ex vivo transfection. Golumbek et al first showed that paracrine delivery of a cytokine at the tumour site could elicit an immune response similar to that of cytokine-modified tumour cells. The authors evaluated the ability of GM-CSF-containing microspheres to act as an adjuvant when mixed with irradiated tumour cells prior to immunization and showed that these preparations produced vaccine effects equivalent to those of GM-CSF-transduced tumour cells (Golumbek et al, 1993) Similarly, vaccines composed of irradiated melanoma cells admixed with liposomal IL-2 are as effective as IL-2 gene modified tumour cells in evoking protective immunity against tumour cells (Koppenhagen et al, 1998). Interestingly, large amounts of free GM-CSF or IL-2 in these same tumour models were ineffective suggesting that sustained release of cytokines at the tumour site was required to achieve the anti-tumour effect (Golumbek et al, 1993; Pardoll, 1995; Koppenhagen et al, 1998). Other successful strategies used for paracrine cytokine delivery at the tumour site have included direct in vivo administration of recombinant viral vectors allowing effective in situ expression of the cytokine gene in the tumour cells or the mix

*Received 14 February 2000*
*Revised 25 July 2000*
*Accepted 9 August 2000*

*Correspondence to:* E Tartour

**Table 1** Patient characteristics

| | Age | Prior treatment | Tumour type | Number of Vero cells administered | Local response | Survival (months) |
|---|---|---|---|---|---|---|
| Patients | | | | | | |
| 1 | 50 | S,R,C,H | Breast adenocarcinoma | $5.10^5$ | NR | >38 |
| 2 | 57 | S,R,C,H | Breast adenocarcinoma | $5.10^5$ | NR | 5 |
| 3 | 52 | S,R,C,H | Breast adenocarcinoma | $5.10^5$ | NR | 27 |
| 4 | 57 | S,R,C,H | Breast adenocarcinoma | $5.10^6$ | HR | >34 |
| 5 | 72 | S,R,C,H | Breast adenocarcinoma | $5.10^6$ | NR | 9 |
| 6 | 65 | S,R,C,H | Breast adenocarcinoma | $5.10^6$ | NR | >25 |
| 7 | 39 | S,R,C,H | Breast adenocarcinoma | $5.10^7$ | NR | >24 |
| 8 | 59 | S,C. | Colorectal carcinoma | $5.10^7$ | HR | 12 |

Abbreviations: S: surgery; R: radiation therapy; C: chemotherapy; H: hormone therapy; NR: no clinical response; HR: local histological response without clinical response.

of cytokine-secreting fibroblasts with tumour cells (Bubenik et al, 1988; Haddada et al, 1993; Addison et al, 1995; Cordier et al, 1995). Various groups have shown that immunization with IL-2 or IL-12-secreting autologous or allogeneic fibroblasts associated with tumour cells induced anti-tumour immunity and was able to cure established tumours in animals (Tahara et al, 1994; Fakhrai et al, 1995; Glick et al, 1997). In man, in a phase I clinical trial, in which autologous tumour cells plus interleukin-2 gene transfected allogeneic fibroblasts were administered to cancer patients, an enhancement of specific anti-tumour T cell responses was observed (Veelken et al, 1997). Roth et al also showed that xenogeneic cells engineered to secrete IL-2 induced protection against tumour development (Roth et al, 1992). In the light of these results, Mehtali et al selected the simian fibroblast Vero cell line as a cell vector for IL-2 production, due to its excellent characterization and safety records established during the pharmaceutical production of human poliovirus vaccine (Leroy et al, 1998). The use of the same IL-2-producing cell line would allow standardization of cytokine production from one patient to another. It also circumvented the risk of long-term persistence in the host of autologous tumour cells or fibroblasts transduced with IL-2 cDNA. Indeed, histoincompatible cells were rejected by the host but, in previous studies, it seemed that the half-life of the IL-2 molecule secreted by these cells was longer than that observed after injection of free recombinant IL-2, which suggests a possible sustained production of cytokine released by histoincompatible cells (Mir et al, 1995).

When a mixture of thyroid medullary carcinoma cells and IL-2-secreting Vero cells was implanted in rats, tumour growth was inhibited as a function of the number of interleukin-secreting Vero cells in the inoculum (Cressent et al, 1995).

Cats with spontaneously arising fibrosarcoma and dogs with buccal melanoma, when treated by tumour surgery, radiotherapy and repeated local injections of xenogeneic cells secreting high levels of IL-2, relapsed less frequently than control animals treated by surgery and radiotherapy alone. In contrast, all cats treated with surgery and irradiation and then injected locally with either recombinant IL-2 or unmodified Vero cells quickly relapsed (Quintin-Colonna et al, 1996). These encouraging data prompted us to conduct a phase I clinical trial, to evaluate the biological and clinical effects of intratumour administration of Vero-IL-2 cells in cancer patients with accessible metastatic tumours.

### PATIENTS AND METHODS

### Patients

Patients with accessible subcutaneous metastases were included in this phase I study. Eligibility criteria also required that patients had not received chemotherapy, radiotherapy or immunotherapy for at least 4 weeks before starting the study and had a predicted survival <3 months and a Karnofsky status index < 70. Other patient characteristics are listed in Table 1.

This study was approved by a regional ethics committee and patients were included after giving their written informed consent.

### Toxicity and response evaluation

A complete history, physical examination, determination of performance status, full blood count, serum biochemistry and urinalysis were obtained before admission into the study for each patient. Serum electrolytes, liver and renal function and blood counts were regularly monitored during each course.

Toxicities were graded on a scale of 0–4 using the Common Toxicity Criteria of the United States National Cancer Institute.

Local clinical response was assessed after each cycle by measuring the largest diameter and the largest perpendicular portion of the nodules in which Vero cells were administered. Tumour evaluation at other metastatic sites included physical examination, chest radiographs and/or computed tomography and ultrasound.

### Preparation and characterization of Vero-IL-2 cells

The monkey Vero fibroblast cell lines were provided by ATCC-CCI81 (Rockville, MD, USA). They were transfected with pTG5324 plasmid containing the human IL-2 cDNA using the calcium phosphate coprecipitation technique and were cultured in DMEM medium with 10% fetal calf serum in the presence of 6 μg $ml^{-1}$ of puromycin (Sigma, Saint Quentin Fallavier, France), as previously described (Quintin-Colonna et al, 1996). Resistant clones were selected and stored in liquid nitrogen in freezing medium (DMEM, 2 mM L-glutamine, 1% non essential amino acids, 0.1% HSA, 5% DMSO) in ready-to-use aliquots of 1 ml containing $3 \times 10^7$ cells. Before administration to the patient, the

© 2000 Cancer Research Campaign

**Table 2** Sequences of 5′ and 3′ primers of 7 target genes

| mRNA species | | Size of PCR products |
|---|---|---|
| β actin | 5′TCG TCG ACA ACG GCT CCG GCA TGT GC | |
| | 3′TTCTGCAGGGAGGAGCTGGAAGCAGC | 688 |
| IFNγ | 5′GGT TCT CTT GGC TGT TAC TGC C | |
| | 3′GTT GGA CAT TCA AGT CAG TTA CCG A | 340 |
| IL-2 End | 5′ACA TGC CCA AGA AGG CCA C | |
| | 3′ AGT GTT GAG ATG ATG CTT TG | 265 |
| IL-2 TG | 5′TCT GAT AGG CAG CCT GCA CC | |
| | 3′TCC TAC AGC TCC TGG GCA ACG | 595 |
| CD3 | 5′TGT CTG AGA GCA GTG TTC CCA C | |
| | 3′CCA GGC TGA TAG TTC GGT GAC C | 220 |
| IL4 | 5′GTA AGC TTC TCC TGA TAA ACT AAT TGC CTC AC | |
| IL4 | 3′AAG AAT TCC AAC GTA CTC TGG TTG GCT TCC TT | 470 |
| IL10 | 5′AGT CTG AGA ACA GCT GCA CCC AC | |
| IL10 | 3′CAC TCA TGG CTT TGT AGA TGC C | 335 |

number of viable cells was determined by Trypan blue dye exclusion. After thawing, the median viability of cells was 86% (range: 70 to 95%). IL-2 production of Vero-IL-2 cells from each batch was also measured using enzyme-linked immunosorbent assay (ELISA) kits purchased from R&D systems (Minneapolis, USA). The median amount of IL-2 produced by injected cells was 130 ng $10^6$ cells$^{-1}$ 24 h ml$^{-1}$ (range: 45–245).

### Study design

Three dose levels of Vero-cells were administered: $5 \times 10^5$, $5 \times 10^6$ and $5 \times 10^7$ cells. Cells were injected into subcutaneous metastatic nodules, three times at 7-day intervals. Three patients were recruited in the first and second dose levels. Only two patients were included in the third dose level.

### Collection of samples

Biopsies of subcutaneous nodules were obtained before and 29 days after intratumour Vero-IL-2 administration. Tissues were divided in two equal parts: one portion was fixed in formalin for histological examination, and the other was snap-frozen in liquid nitrogen and stored at –70°C for RNA extraction. Vero-IL-2 cells were injected into a subcutaneous nodule adjacent to that chosen for pretreatment biopsy.

Plasma for cytokine assay and whole blood for vector detection were collected from all patients before treatment and on days 3, 8, 10, 15, 17, 22 after the first administration of Vero IL-2 cells.

### Assays

IL-2 and IL-4 serum levels were determined by commercially available ELISA kits from R&D (Minneapolis, MN). Soluble IL-2 receptor was measured using ELISA kits from Immunotech (Marseille, France). IFNγ was assayed using an ELISA kit from Diaclone (Besançon, France).

### Cytokine mRNA analysis in tumour biopsies

Total cellular RNA was extracted from peripheral whole blood using the RNA-zol B method. Two µg of total cellular RNA were mixed with oligo-dT primers to synthesize cDNA (First strand cDNA synthesis kit; Boehringer-Mannheim, Germany) according to the manufacturer's protocol.

PCR was performed as previously described (Tartour et al, 1998). Table 2 gives the sequence of cytokine primers used in this study and the design of the two pairs of IL-2 primers chosen to discriminate between endogenous IL-2 and exogenous IL-2.

The size of the PCR products will allow us to discriminate between endogenous and exogenous IL-2 mRNA.

### Histochemical analysis

For every patient, paraffin-embedded sections derived from pretreatment and post-treatment biopsies were stained with haematoxylin and eosin. All histological sections were reviewed by two pathologists.

### RESULTS

### Clinical findings

### *Toxicity*

The treatment and injection procedures were well tolerated. No laboratory abnormality and no sign of organ toxicity related to treatment were recorded in any of the 8 patients analysed. No sign related to the IL-2 toxicity previously observed when administered systemically (hypotension, renal dysfunction, weight gain, haematological disorder, nausea) was recorded.

During the course of treatment, patient 2 developed cholestasis secondary to disease progression with bile duct compression.

### *Clinical response and follow-up*

No patient exhibited any clinical regression of the treated subcutaneous nodules. Patients 3 and 4 developed local erythema and inflammation at the site of the injected nodules 48 hours after the start of therapy during the first cycle for patient 3 and the third cycle for patient 4. This local phenomenon resolved spontaneously. Patients 4 and 7 were considered to present stable disease 3 months after the start of therapy, while the other patients clinically progressed. Survival analysis is described for each patient in Table 1.

 *© 2000 Cancer Research Campaign*

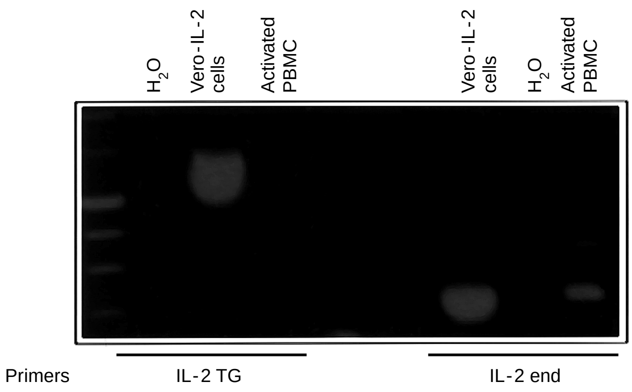

**Figure 1** Design of primers to discriminate endogenous and exogenous IL-2 mRNA. RT-PCR was performed on mRNA extracted from Vero-IL-2 cells or PHA-activated peripheral blood mononuclear cells (PBMC). Two couples of primers were used: IL-2 TG, which specifically amplifies the sequence of IL-2 cDNA included in the pTG5324 plasmid, whereas IL-2 end detected both exogenous and endogenous IL-2. Amplified products were loaded onto 2% agarose gel and stained with ethidium bromide. Negative controls in which $H_2O$ replaced cDNA were also included.

### Laboratory findings

### *Vector detection in peripheral whole blood and tumour specimens*

We designed primers to discriminate between endogenous IL-2 mRNA and exogenous IL-2 mRNA derived from Vero-IL-2 cells. Figure 1 shows that IL-2 TG primers amplified cDNA prepared from Vero-cell mRNA, but not from activated peripheral whole blood (PBMC) mRNA, whereas IL-2 end primers detected mRNA derived from both Vero-IL-2 cells and activated PBMC. IL-2 TG primers were unable to detect exogenous IL-2 mRNA – a surrogate marker of the persistence of Vero-IL-2 cells – in peripheral whole blood of any of the patients. Samples were collected before each cycle and 48 and 96 hours after Vero-IL-2 cell injection in tumour cells. Using the same technique, we did not find any expression of exogenous IL-2 mRNA derived from Vero-cells in post-treatment tumour biopsies removed 29 days after the start of therapy. However, we could not accurately assess the half life of the Vero cells at the tumour site.

### *Histochemical analysis of pre and post-treatment biopsies*

Post-treatment biopsies of patients 4 and 8 showed complete disappearance of tumour cells, which were replaced by granuloma and inflammatory cells (Figure 2A, B and data not shown). This

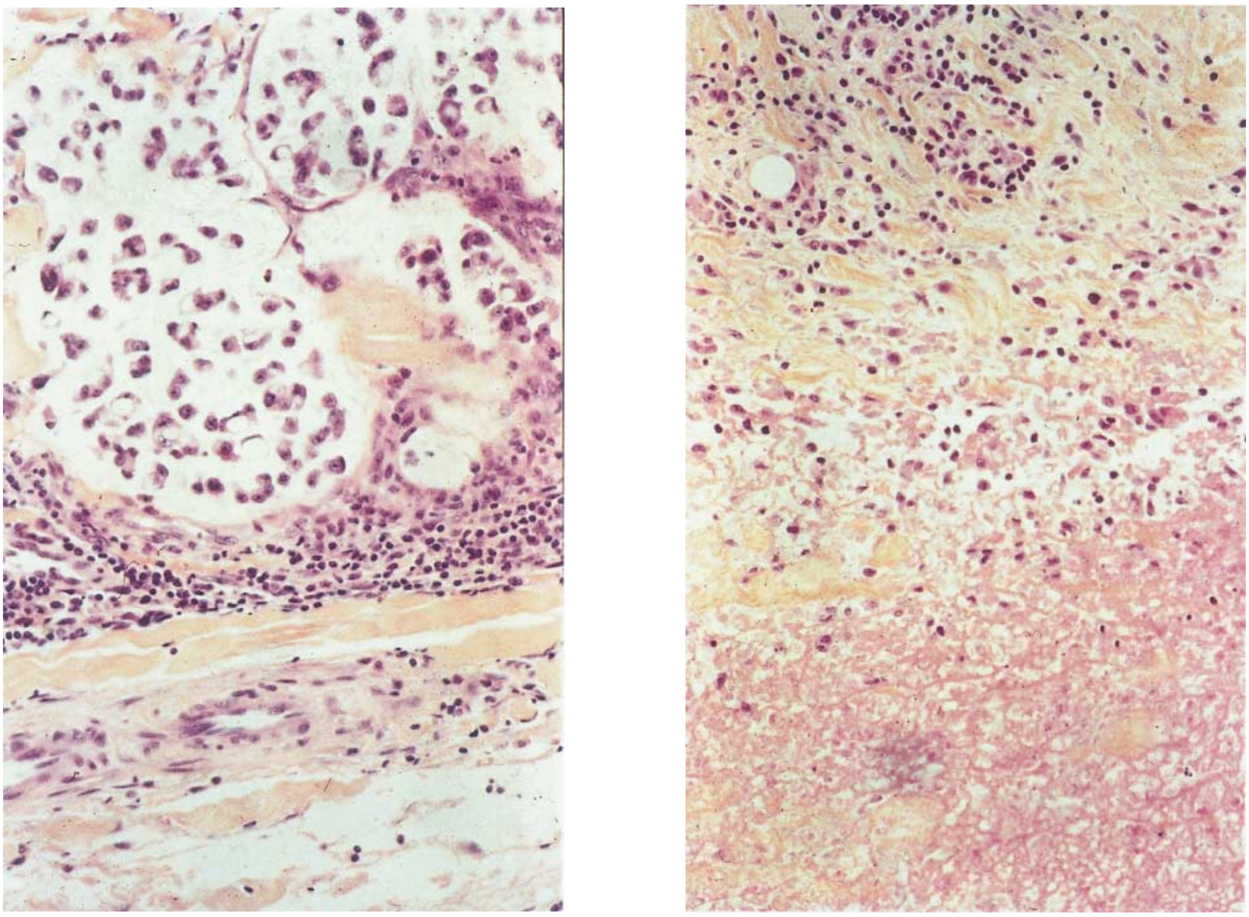

**Figure 2** Histological analysis of pre- and post-treatment nodules of patient 4. (Magnification × 40). Paraffin-embedded sections were stained with haematoxylin and eosin. (**A**) Pre-treatment biopsies: adenocarcinoma cells were encapsulated and surrounded by infiltrating cells. (**B**) Post-treatment biopsies performed 29 days after Vero-IL-2 cells administration. Complete disappearance of tumour cells which were replaced by granuloma and inflammatory cells.

© 2000 Cancer Research Campaign

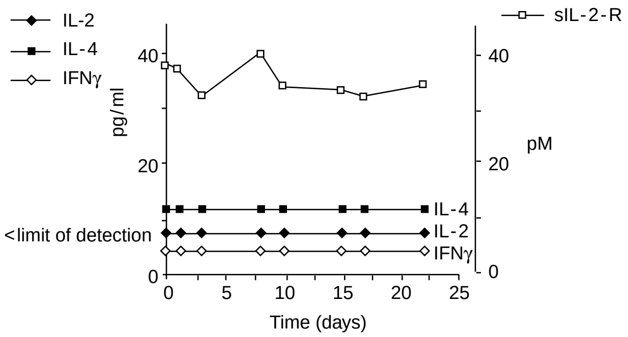

**Figure 3** Cytokine detection in plasma of patient 1. Soluble IL-2 receptor (sIL-2-R), IL-2, IL-4 and IFNγ were measured in the plasma of patient 1 before and at different times after intratumour Vero-IL-2 cell administration. sIL-2R scale (pM) is shown on the right of the figure, whereas IL-2, IL4 and IFNγ scale (pg ml$^{-1}$) is shown on the left of the figure. The results shown are representative of the profile observed in the series of patients. IL-2, IL-4 and IFNγ levels were below the limit of detection.

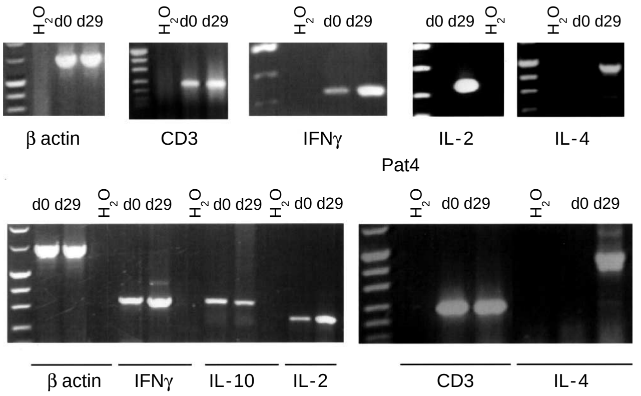

**Figure 4** Cytokine mRNA determination in pre- and post-treatment biopsies derived from patients with histological response (patients 4 and 8). mRNA derived from pre-treatment nodules (d0) and post-treatment nodules (d29) were reverse transcribed into cDNA. A PCR was then performed using different primers specific for CD3, IFNγ, IL-2 and IL-4 cDNA. For patient 8, specific primers for IL-10 were also introduced. Amplified products were loaded onto 2% agarose gel and stained with ethidium bromide. All results were normalized to β actin gene expression. Negative controls in which $H_2O$ replaced cDNA were also included.

local histological response of treated nodules was therefore not expected on the basis of clinical findings.

To assess the possible relationship between this histological response and Vero-IL-2 cell administration, one non-treated nodule was removed in patients 4 and 8. Metastatic tumour cells were found in all biopsies analysed with no significant inflammatory reaction. These results support the hypothesis that the local clinical anti-tumour response was related to Vero-IL-2 therapy. They also suggest that the host reaction remained localized to the site of Vero-IL-2 cell injection.

### *Cytokine determination in serum and biopsies of patients*

IL-2 has been shown to induce a cascade of cytokines in vivo which could reflect its in vivo activity (Gemlo et al, 1988; Schaafsma et al, 1991; Tartour et al, 1992). Therefore, to address a possible systemic effect of intra-tumour administration of Vero cells, cytokines were measured in the plasma of cancer patients before and after local therapy. We did not find an increase of serum

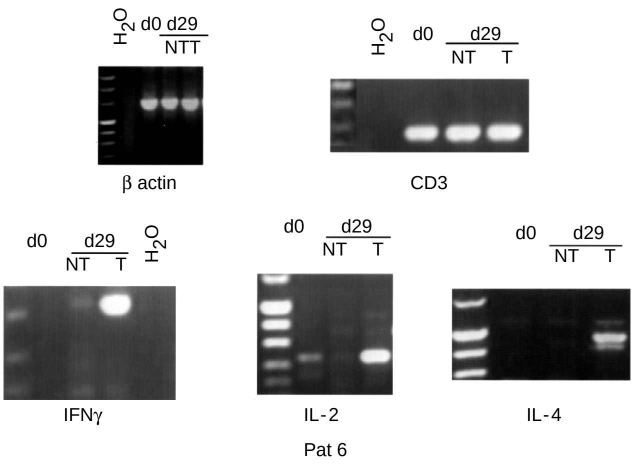

**Figure 5** Cytokine mRNA determination in Vero-IL-2 cell treated and non-treated nodules of patient 6. mRNA derived from pre-treatment nodules (d0) and post-treatment treated (T) or non-treated (NT) nodules (d29) were reverse transcribed into cDNA. A PCR was then performed using different primers specific for CD3, IFNγ, IL-2 and IL-4 cDNA. Amplified products were loaded onto 2% agarose gel and stained with ethidium bromide. All results were normalized to β actin gene expression.

IL-2, IL-4 and IFN-γ levels for any patient on samples collected before treatment and on days 3, 8, 10, 15, 17 and 22 after administration of Vero cells (Figure 3 and data not shown). Previous studies reported that the presence of IL-2 in the systemic circulation is often very transient, even after i.v. IL-2 administration and that the increase of serum soluble IL-2 receptor levels is more stable and easier to monitor (Lotze et al, 1987; Konrad et al, 1990). We, therefore, also measured serum sIL-2R levels in the patients. We did not find any significant induction of sIL-2R levels after local injection of Vero-IL-2 cells, which supports the absence of a marked systemic release of IL-2 from the tumour site of injection. These results are in line with those reported above concerning the absence of exogenous IL-2 mRNA detected in the circulation of treated patients.

Cytokines are considered to exert their biological effect via a paracrine mechanism (Pardoll, 1995). To assess a possible local in vivo biological effect of this cell therapy, we established a cytokine mRNA profile in biopsies taken from patients before and 1 month after Vero-IL-2 cell injection. For this semi-quantitative analysis, all results were normalized to β actin gene expression.

Marked induction of IFNγ, IL-2 and IL-4 mRNA was demonstrated in post-treatment biopsies of patients 1, 3, 4, 6, and 8 (Figures 4, 5 and data not shown). Two of these patients (4 and 8) exhibited a histological response of the treated nodules.

Patient 3 developed local erythema 48 hours after the first injection of Vero-IL-2 cells. It should be emphasized that, in contrast with the rather specific T and NK cell-derived cytokines (IL-2, IL-4, IFNγ), IL-10 mRNA was not found to be increased in post-treatment biopsies of patient 8 (Figure 4). In an attempt to relate this local cytokine activation to treatment, treated and non-treated nodules were both removed 29 days after therapy in patient 6. The activation state was only observed in the treated nodule (Figure 5).

In some patients (1, 6, 8), this increase was not associated with a parallel increase of CD3 mRNA, a T cell marker (Figures 4, 5 and data not shown).

 *© 2000 Cancer Research Campaign*

### DISCUSSION

No severe adverse effect was observed in the 8 patients analysed during this phase I clinical trial. The maximal tolerated dose (MTD) was therefore not reached even in the higher dose group. This absence of toxicity seems to be associated with rapid elimination of Vero-IL-2 cells from the body as exogenous IL-2 mRNA could no longer be detected in peripheral whole blood collected as early as 48 hours after Vero-IL-2 cell administration and in the patients' post-treatment biopsies.

These results are in line with previous studies in which a positive transgene DNA signal in the biopsies was demonstrated only 1 hour after intra-tumour injection of Vero-IL-2 cells in cats (Quintin-Colonna et al, 1996). In a similar phase I clinical trial, Rochlitz also did not find any expression of exogenous mRNA 24 hours after local cell injection (Rochlitz et al, 1999). As expected, these data suggest that xenogenic cells are rapidly rejected by the host.

One major finding of this trial concerns the histological response without clinical response of two treated subcutaneous nodules in two patients. The relationship between local treatment and tumour regression was reinforced by the replacement of tumour cells by inflammatory cells in regressing lesions. Furthermore, different non-treated control nodules collected at the same time period after therapy in these two patients did not exhibit any significant inflammatory reaction and tumour cells were present in all cases.

This discrepancy between clinical and histological results was also reported in a series of melanoma patients treated by IFNα and high-dose IL-2, as in 11 patients, residual tumour lesions were resected and histology revealed almost complete tumour regression in 6 patients (Keilholz et al, 1994). Clinical evaluation during immunotherapy protocols may therefore underestimate the anti-tumour effect of these biological response modifiers.

This peculiar histological response was associated with marked induction of T and NK cell-derived cytokines (IL-2, IL-4, IFNγ…) in post-treatment lesions. This intra-tumour cytokine induction was also observed in 3 non-responder patients. This finding is reminiscent of previous results, in which activation of immune system was a necessary but not sufficient condition to achieve clinical response (Dorval et al, 1992; Tartour et al, 1992). In order to correlate this activation state to treatment, we showed that no marked induction of IL-2, IL-4 or IFNγ mRNA could be demonstrated in non-treated control biopsies (Figure 5).

Although the antitumour effect of IL-2 and IFNγ has been clearly established, the clinical significance of IL-4 is more controversial. Pioneer studies in murine models clearly associated the increase of IL-4 mRNA in the spleen of tumour-bearing mice with tumour progression (Ghosch et al, 1995). In man, an increase of TH2 cytokines (IL-4, IL-10) with a defect of TH1 cytokine (IL-2, IFNγ) at the tumour site has been reported in several models (Wang et al, 1995; Chouaib et al, 1997; Tartour et al, 1998). In some circumstances immunotherapy can correct this defect (Vowels et al, 1994). On the basis of these observations, IL-4 was therefore considered to be a marker of poor prognosis. However, other studies in murine models showed that tumours transfected with IL-4 cDNA were rejected and could protect against a challenge with parental tumour (Tepper et al, 1989; Golumbek et al, 1991). In vivo delivery of interleukin-4 by a recombinant vaccinia virus prevented tumour development in mice (Elkins et al, 1994). Recently, Schuler et al demonstrated that IL-4 knock out mice were severely impaired to develop tumour immunity (Schuler et al, 1999). This lack of tumour immunity in IL-4 knock out mice was associated with reduced IFNγ production and undetectable CTL activity. The IL-4 mRNA induction observed in tumour biopsies after Vero cells administration may therefore be associated with a clinical benefit.

The design of the protocol did not allow us to assess the respective roles of xenogenic cells and IL-2. However, in murine models, histoincompatible cells not secreting IL-2 only display marginal effects on tumour growth (Roth et al, 1992). Cats treated by surgery, radiotherapy and repeated local injections of xenogeneic Vero cells not secreting IL-2 relapsed more frequently than when Vero cells secreting high levels of IL-2 were used (Quintin-Colonna et al, 1996)

The rationale of this immunotherapy approach is discussed by several groups, as it has been shown that local IL-2 production is most effective when the helper cytokine is secreted by the tumour cell (Tsai et al, 1993). The use of allogeneic fibroblasts or allogeneic tumour cells modified to secrete cytokines has been found to be less potent to induce an antitumour response than when syngenic fibroblasts or autologous tumour cells transfected with IL-2 were used as a vaccine (Aruga et al, 1997; Bowman et al, 1998). Allogeneic or xenogeneic cells engineered to secrete IL-2 induce protection against tumour development via stimulation of IL-2-dependent natural killer activity, whereas syngenic tumour cells transfected with IL-2 confer protection by stimulating tumour-specific CTL (Roth et al, 1992; Tsai et al, 1993). This may explain the absence of clinical or immunological effects observed at sites distant from the injection of Vero IL-2 cells.

Melanoma and renal cell carcinoma are usually considered to be the main tumours sensitive to immunotherapy (Mukherji and Chakraborty, 1995). In this trial and a similar trial reported by Rochlitz et al (1999), colorectal carcinoma, sarcoma and breast carcinoma tumour cells appeared to be sensitive to this type of immunotherapy. The safety of this treatment procedure associated with these promising data prompted us to initiate a multicentre phase II trial.

### ACKNOWLEDGEMENTS

This work was supported by Institut Curie, INSERM, Université Pierre and Marie Curie and grants from Ligue Nationale contre le Cancer and Indo-French Centre for the Promotion of Advanced Research (IFCPAR).

### REFERENCES

Addison CL, Braciak T, Ralston R, Muller WJ, Gauldie J and Graham FL (1995) Intratumoral injection of an adenovirus expressing interleukin 2 induces regression and immunity in a murine breast cancer model. *Proc Natl Acad Sci USA* **92**: 8522–8526

Aruga A, Aruga E and Chang AE (1997) Reduced efficacy of allogeneic versus syngeneic fibroblasts modified to secrete cytokines as a tumor vaccine adjuvant. *Cancer Res* **57**: 3230–3237

Berd D, Kairys J, Dunton C, Mastrangelo MJ, Sato T and Maguire HC Jr (1998) Autologous, hapten-modified vaccine as a treatment for human cancers. *Semin Oncol* **25**: 646–653

Bowman LC, Grossmann M, Rill D, Brown M, Zhong WY, Alexander B, Leimig T, Coustan-Smith E, Campana D, Jenkins J, Woods D and Brenner M (1998) Interleukin-2 gene-modified allogeneic tumor cells for treatment of relapsed neuroblastoma. *Human Gene Ther* **9**: 1303–1311

© 2000 Cancer Research Campaign

Bubenik J, Voitenok NN, Kieler J, Prassolov VS, Chumakov PM, Bubenikova D, Simova J and Jandlova T (1988) Local administration of cells containing an inserted IL-2 gene and producing IL-2 inhibits growth of human tumours in nu/nu mice. *Immunol Letters* **19**: 79–282

Chen L (1998) Immunological ignorance of silent antigens as an explanation of tumor evasion. *Immunol Today* **19**: 27–30

Chouaib S, Asselin-Paturel C, Mami-Chouaib F, Caignard A and Blay JY (1997) The host-tumor immune conflict: from immunosuppression to resistance and destruction. *Immunol Today* **18**: 493–497

Clary BM, Coveney EC, Philip R, Blazer DG 3rd, Morse M, Gilboa E and Lyerly HK (1997) Inhibition of established pancreatic cancers following specific active immunotherapy with interleukin-2 gene-transduced tumor cells. *Cancer Gene Ther* **4**: 97–104

Cordier L, Duffour MT, Sabourin JC, Lee MG, Cabannes J, Ragot T, Perricaudet M and Haddada (1995) Complete recovery of mice from a pre-established tumor by direct intratumoral delivery of an adenovirus vector harboring the murine IL-2 gene. *Gene Ther* **2**: 16–21

Cressent M, Pidoux E, Cohen R, Modigliani E and Roth C (1995) Interleukin-2 and interleukin-4 display potent antitumour activity on rat medullary thyroid carcinoma cells. *Eur J Cancer* **31A**: 2379–2384

Dorval T, Mathiot C, Chosidow O, Revuz J, Avril MF, Guillaume JC, Tursz T, Brandely M, Pouillart P and Fridman WH (1992) IL-2 phase II trial in metastatic melanoma: analysis of clinical and immunological parameters. *Biotec Therap* **3**: 63–79

Dow W, Elmslie RE, Willson AP, Roche L, Gorman C and Potter TA (1998) In vivo tumor transfection with superantigen plus cytokine genes induces tumor regression and prolongs survival in dogs with malignant melanoma. *J Clin Invest* **101**: 2406–2414

Dranoff G, Jaffee E, Lazenby A, Golumbek P, Levitsky H, Brose K, Jackson V, Hamada H, Pardoll D and Mulligan RC (1993) Vaccination with irradiated tumor cells engineered to secrete murine granulocyte-macrophage colony-stimulating factor stimulates potent, specific, and long-lasting anti-tumor immunity. *Proc Natl Acad Sci USA* **90**: 3539–3543

Elkins KL, Ennist DL, Winegar RK and Weir JP (1994) In vivo delivery of interleukin-4 by a recombinant vaccinia virus prevents tumor development in mice. *Human. Gene. Ther* **5**: 809–820

Fakhrai H, Shawler DL, Gjerset R, Naviaux RK, Koziol J, Royston I and Sobol RE (1995) Cytokine gene therapy with interleukin-2-transduced fibroblasts: effects of IL-2 dose on anti-tumor immunity. *Human Gene Ther* **6**: 591–601

Fearon ER, Itaya T, Hunt B, Vogelstein B and Frost P (1988) Induction in a murine tumor of immunogenic tumor variants by transfection with a foreign gene. *Cancer Res* **48**: 2975–2980

Fearon ER, Pardoll DM, Itaya T, Golumbek P, Levitsky HI, Simons JW, Karasuyama H, Vogelstein B and Frost P (1990) Interleukin-2 production by tumor cells bypasses T helper function in the generation of an antitumor response. *Cell* **60**: 397–403

Gansbacher B, Zier K, Daniels B, Cronin K, Bannerji R and Gilboa E (1990) Interleukin 2 gene transfer into tumor cells abrogates tumorigenicity and induces protective immunity. *J Exp Med* **172**: 1217–1224

Gemlo BT, Palladino MA Jr, Jaffe HS, Espevik TP and Rayner AA (1988) Circulating cytokines in patients with metastatic cancer treated with recombinant interleukin 2 and lymphokine-activated killer cells. *Cancer Res* **48**: 5864–5867

Ghosh P, Komschlies KL, Cippitelli M, Longo DL, Subleski J, Ye J, Sica A, Young HA, Wiltrout RH and Ochoa AC (1995) Gradual loss of T-helper 1 populations in spleen of mice during progressive tumor growth. *J Natl Cancer Inst* **87**: 1478–1483

Glick RP, Lichtor T, Mogharbel A, Taylor CA and Cohen EP (1997) Intracerebral versus subcutaneous immunization with allogeneic fibroblasts genetically engineered to secrete interleukin-2 in the treatment of central nervous system glioma and melanoma. *Neurosurgery* **41**: 898–906

Golumbek PT, Lazenby AJ, Levitsky HI, Jaffee LM, Karasuyama H, Baker M and Pardoll DM (1991) Treatment of established renal cancer by tumor cells engineered to secrete interleukin-4. *Science* **254**: 713–716

Golumbek PT, Azhari R, Jaffee EM, Levitsky HI, Lazenby A, Leong K and Pardoll DM (1993) Controlled release, biodegradable cytokine depots: a new approach in cancer vaccine design. *Cancer Res* **53**: 5841–5844

Haddada H, Ragot T, Cordier L, Duffour MT and Perricaudet M (1993) Adenoviral interleukin-2 gene transfer into P815 tumor cells abrogates tumorigenicity and induces antitumoral immunity in mice. Human. *Gene Ther* **4**: 703–711

Heo DS, Yoon SJ, Kim WS, Lee KH, Seol JG, Lee SG, Jung CW, Cho EK, Kim CW, Park MH, Sung MW, Kim KH, Bang YJ and Kim NK (1998) Locoregional response and increased natural killer activity after intratumoral injection of HLA-B7/beta2 microglobulin gene in patients with cancer. *Human Gene Ther* **9**: 2031–2038

Janeway CA Jr and Bottomly K (1994) Signals and signs for lymphocyte responses. *Cell* **76**: 275–285

Keilholz U, Scheibenbogen C, Stoelben E, Saege RHD and Hunstein W (1994) Immunotherapy of metastatic melanoma with interferon-alpha and interleukin-2: pattern of progression in responders and patients with stable disease with or without resection of residual lesions. *Eur J Cancer* **30A**: 955–958

Konrad MW, Hemstreet G, Hersh EM, Mansell PW, Mertelsmann R, Kolitz JE and Bradley EC (1990) Pharmacokinetics of recombinant interleukin 2 in humans. *Cancer Res* **50**: 2009–2017

Koppenhagen FJ, Kupcu Z, Wallner G, Crommelin DJ, Wagner E, Storm G and Kircheis R (1998) Sustained cytokine delivery for anticancer vaccination: liposomes as alternative for gene-transfected tumor cells. *Clin Cancer Res* **4**: 1881–1886

Leroy P, Slos P, Homann H, Erbs P, Poitevin Y, Regulier E, Colonna FQ, Devauchelle P, Roth C, Pavirani A and Mehtali M (1998) Cancer immunotherapy by direct in vivo transfer of immunomodulatory genes. *Res Immunol* **149**: 681–684

Lindenmann J and Klein PA (1967) Viral oncolysis: increased immunogenicity of host cell antigen associated with influenza virus. *J Exp Med* **126**: 93–108

Lotze MT, Custer MC, Sharrow SO, Rubin LA, Nelson DL and Rosenberg SA (1987) In vivo administration of purified human interleukin-2 to patients with cancer: development of interleukin-2 receptor positive cells and circulating soluble interleukin-2 receptors following interleukin-2 administration. *Cancer Res* **47**: 2188–2195

Maass G, Schmidt W, Berger M, Schilcher F, Koszik F, Schneeberger A, Stingl G, Birnstiel ML and Schweighoffer T (1995) Priming of tumor-specific T cells in the draining lymph nodes after immunization with interleukin 2-secreting tumor cells: three consecutive stages may be required for successful tumor vaccination. *Proc Natl Acad Sci USA* **92**: 5540–5544

Mir LM, Roth C, Orlowski S, Quintin-Colonna F, Fradelizi D, Belehradek J Jr and Kourilsky P (1995) Systemic antitumor effects of electrochemotherapy combined with histoincompatible cells secreting interleukin-2. *J Immunother* **17**: 30–38

Mukherji B and Chakraborty NG (1995) Immunobiology and immunotherapy of melanoma. *Curr Opin Oncol* **7**: 175–184

Pardoll DM (1995) Paracrine cytokine adjuvants in cancer immunotherapy. *Annu Rev Immunol* **13**: 399–415

Plautz GE, Yang ZY, Wu BY, Gao X, Huang L and Nabel GJ (1993) Immunotherapy of malignancy by in vivo gene transfer into tumors. *Proc Natl Acad Sci US*A **90**: 4645–4649

Quintin-Colonna F, Devauchelle P, Fradelizi D, Mourot B, Faure F, Kourilsky P, Roth C and Mehtali M (1996) Gene therapy of spontaneous canine melanoma and feline fibrosarcoma by intratumoral administration of histoincompatible cells expressing human interleukin-2. *Gene Ther* **3**: 1104–1112

Rochlitz C, Jantscheff P, Bongartz G, Dietrich PY, Quiquerez AL, Schatz C, Mehtali M, Courtney M, Tartour E, Dorval T, Fridman WH and Herrmann R (1999) Gene therapy study of cytokine-transfected xenogeneic cells (Vero-interleukin-2) in patients with metastatic solid tumors. *Cancer Gene Ther* **6**: 271–281

Roth C, Mir LM, Cressent M, Quintin-Colonna F, Ley V, Fradelizi D and Kourilsky P (1992) Inhibition of tumor growth by histoincompatible cells expressing interleukin-2. *Int Immunol* **4**: 1429–1436

Schaafsma MR, Falkenburg JH, Landegent JE, Duinkerken N, Osanto S, Ralph P, Kaushansky K, Wagemaker G, Van Damme J, Willemze R and Fibbe WE (1991) In vivo production of interleukin-5, granulocyte-macrophage colony-stimulating factor, macrophages colony-stimulating factor, and interleukin-6 during intravenous administration of high-dose interleukin-2 in cancer patients. *Blood* **78**: 1981–1987

Schmidt-Wolf GD and Schmidt-Wolf IG (1995) Cytokines and gene therapy. *Immunol Today* **16**: 173–175

Schuler T, Qin Z, Ibe S, Noben-Trauth N and Blankenstein T (1999) T helper cell type 1-associated and cytotoxic T lymphocyte-mediated tumor immunity is impaired in interleukin 4-deficient mice. *J Exp Med* **189**: 803–810

Speiser DE, Miranda R, Zakarian A, Bachmann MF, McKall-Faienza K, Odermatt B, Hanahan D, Zinkernagel RM and Ohashi PS (1997) Self antigens expressed by solid tumors Do not efficiently stimulate naive or activated T cells: implications for immunotherapy. *J Exp Med* **186**: 645–653

Stopeck AT, Hersh EM, Akporiaye ET, Harris DT, Grogan T, Unger E, Warneke J, Schluter SF and Stahl S (1997) Phase I study of direct gene transfer of an

 *© 2000 Cancer Research Campaign*

allogeneic histocompatibility antigen, HLA-B7, in patients with metastatic melanoma. *J Clin Oncol* **15**: 341–349

Tahara H, Zeh HJ 3rd, Storkus WJ, Pappo I, Watkins SC, Gubler U, Wolf SF, Robbins PD and Lotze MT (1994) Fibroblasts genetically engineered to secrete interleukin 12 can suppress tumor growth and induce antitumor immunity to a murine melanoma in vivo. *Cancer Res* **54**: 182–189

Tartour E, and Fridman WH (1998) Cytokines and cancer. *Int Rev Immunol* **16**: 683–704

Tartour E, Mathiot C and Fridman WH (1992) Current status of interleukin-2 therapy in cancer. *Biomed Pharmacother* **46**: 473–484

Tartour E, Gey A, Sastre-Garau X, Lombard Surin I, Mosseri V and Fridman WH (1998) Prognostic value of intratumoral interferon gamma messenger RNA expression in invasive cervical carcinomas. *J Natl Cancer Inst* **90**: 287–294

Tepper RI, Pattengale PK and Leder P (1989) Murine interleukin-4 displays potent anti-tumor activity in vivo. *Cell* **57**: 503–512

Tsai SC, Gansbacher B, Tait L, Miller FR and Heppner GH (1993) Induction of antitumor immunity by interleukin-2 gene-transduced mouse mammary tumor cells versus transduced mammary stromal fibroblasts. *J Natl Cancer Inst* **85**: 546–553

Veelken H, Mackensen A, Lahn M, Kohler G, Becker D, Franke B, Brennscheidt U, Kulmburg P, Rosenthal FM, Keller H, Hasse J, Schultze-Seemann W, Farthmann EH, Mertelsmann R and Lindemann A (1997) A phase-I clinical study of autologous tumor cells plus interleukin-2-gene-transfected allogeneic fibroblasts as a vaccine in patients with cancer. *Int J Cancer* **70**: 269–277

Vowels BR, Lessin SR, Cassin M, Jaworsky C, Benoit B, Wolfe JT and Rook AH (1994) Th2 cytokine mRNA expression in skin in cutaneous T-cell lymphoma. *J Invest Dermatol* **103**: 669–673

Wang Q, Stanley J, Kudoh S, Myles J, Kolenko V, Yi T, Tubbs R, Bukowski R and Finke J (1995) T cells infiltrating non-Hodgkin's B cell lymphomas show altered tyrosine phosphorylation pattern even though T cell receptor/CD3-associated kinases are present. *J Immunol* **155**: 1382–1392

Zatloukal K, Schneeberger A, Berger M, Schmidt W, Koszik F, Kutil R, Cotten M, Wagner E, Buschle M, Maass G, Payer E, Stingl G and Birnstiel ML (1995) Elicitation of a systemic and protective anti-melanoma immune response by an IL-2-based vaccine. Assessment of critical cellular and molecular parameters *J Immunol* **154**: 3406–3419

*© 2000 Cancer Research Campaign*
